# Directional Plasmonic Excitation by Helical Nanotips

**DOI:** 10.3390/nano11051333

**Published:** 2021-05-19

**Authors:** Leeju Singh, Nicolò Maccaferri, Denis Garoli, Yuri Gorodetski

**Affiliations:** 1 Electrical and Electronics Engineering Department, Ariel University, Ariel 40700, Israel; leejule@ariel.ac.il; 2 Department of Physics and Materials Science, University of Luxembourg, 162a avenue de la Faïencerie, L-1511 Luxembourg, Luxembourg; nicolo.maccaferri@uni.lu; 3 Istituto Italiano di Tecnologia, Via Morego 30, 16163 Genova, Italy; 4 Libera Università di Bolzano, Piazza Università 1, 39100 Bolzano, Italy; 5 Mechanical Engineering and Mechatronics Department, Ariel University, Ariel 40700, Israel

**Keywords:** plasmonics, nanotip, chiral, symmetry breaking, directional excitation

## Abstract

The phenomenon of coupling between light and surface plasmon polaritons requires specific momentum matching conditions. In the case of a single scattering object on a metallic surface, such as a nanoparticle or a nanohole, the coupling between a broadband effect, i.e., scattering, and a discrete one, such as surface plasmon excitation, leads to Fano-like resonance lineshapes. The necessary phase matching requirements can be used to engineer the light–plasmon coupling and to achieve a directional plasmonic excitation. Here, we investigate this effect by using a chiral nanotip to excite surface plasmons with a strong spin-dependent azimuthal variation. This effect can be described by a Fano-like interference with a complex coupling factor that can be modified thanks to a symmetry breaking of the nanostructure.

## 1. Introduction

Fano interference is a well-known physical phenomenon that occurs when two oscillating systems interact, one of which is characterized by a narrow resonance and the other having a broadband response [1,2,3]. In such a case, an asymmetric lineshape of the resonance with respect to the driving force frequency [4] should appear. The Fano effect has recently received a lot of attention as its different implementations have been demonstrated in a number of physical systems [1,3,5]. Systems involving scattering and plasmonic excitations are of particular interest in photonics [4,6,7]. The coupling strength of the two systems can vary due to the intensity ratio and the relative phase of the continuum and discrete function [8,9]. By varying this coupling factor, the couple system can be driven into an anti-resonant state, where the total response is fully suppressed due to a destructive interference [10]. As a result, the plasmonic wave excitation becomes extremely sensitive to a phase lag, and unexpected asymmetry in wave front propagation can occur. The Fano lineshape tuning in nanostructures by complex phase matching conditions was recently demonstrated by using circularly polarized light impinging on a subwavelength scatterer to excite a radially propagating plasmonic wave [11,12]. The azimuthal variance in the k-space revealed a high SP directionality when the scatterer was slightly shifted in the lateral direction. It has been shown that this directionality was strongly dependent on the handedness of the circular polarization state—the incident spin.

A model for this spin-dependent directionality has been also reported [12,13], where the azimuthally varying Fano coupling is originated from the spin–orbit interaction of the tightly focused beam at the subwavelength scatterer [14]. The coupling mechanism can be represented by a complex coupling factor, between the excitation light configuration and the scatterer geometry. While this phenomenon has been demonstrated for the lateral displacement of the structure from the optical axis, here we would like to experimentally investigate this effect exploring the interference between circularly polarized light and a scatterer—where the symmetry of the scatterer itself is broken. In order to do that, helical nanostructures were proposed. In particular, different three-dimensional helical metallic structures have been recently investigated as chiral metamaterials for advanced nanophononics [15,16].

Here, we used our robust fabrication method [17,18] to prepare high aspect ratio metallic nanotips with integrated spiral corrugations in the tip’s body. These helical tips can integrate spirals with different topological configurations. In particular, as extensively demonstrated, the use of Archimede’s spirals with different number of arms (*m*) enables one to play with the spin–orbit coupling between the impinging light and the generated surface plasmon polaritons (SPPs) [17,18,19,20,21,22]. We investigated how the proposed tips can be used to achieve a spin–orbit control of the directional excitation of surface plasmons [23,24,25,26,27,28]. We used leakage microscopy [29,30] to probe the k-space in the imaging system. This enables to directly observe the excited surface plasmons and the dependency of the plasmon polaritons propagation on the chirality of the structure and the used polarization of the impinging light.

## 2. Materials and Methods

### 2.1. Nanotip Fabrication

The fabrication of the samples is based on a procedure described in several recent papers [17,18]. The principle relies on the FIB-generated secondary-electron lithography in optical resist, and allows the preparation of high aspect ratio structure with any 3D profile. The final structure comprises a 5 µm high base-smoothed gold tip with a tip’s apex curvature about 50 nm. The tip is prepared on a transparent substrate (100 nm thick Si_3_N_4_ membrane) and coated with a thin gold layer. The skeleton of the tip is made of S1813 optical resist exposed with secondary electron during its milling to create the desired shape. With respect to the previously reported tip fabrication [17,18], here we introduced an additional step where the smooth tip shape is finalized with an embedded Archimede’s spiral with radius R(φ) and *m* arms according to the following equation:

(1) $$R_{m}\left(\varphi \right)=R_{0}+m\varphi /k_{SP}$$

where φ is the azimuthal angle and $\;k_{SP}=2\pi /\lambda_{sp}$ is the plasmonic wavenumber, where $\lambda_{SP}$ is the wavelength of the SPP mode on a flat gold–air interface. After the exposure, the obtained dielectric tip is coated with a 60 nm thick gold layer in order to keep it sufficiently transparent for the leakage microscopy characterization. Considering that we are interested in the propagating plasmon polaritons, the base of the tip (partially transparent) was back-filled with localized deposition of platinum by using an electron beam induced deposition. To prove the concept, we have fabricated 2 different helical tips with topological numbers m=±1,  ±3 and a bare tip without a spiral groove.

### 2.2. Leakage Microscopy for k-Space Microscopy

**Figure 1:**
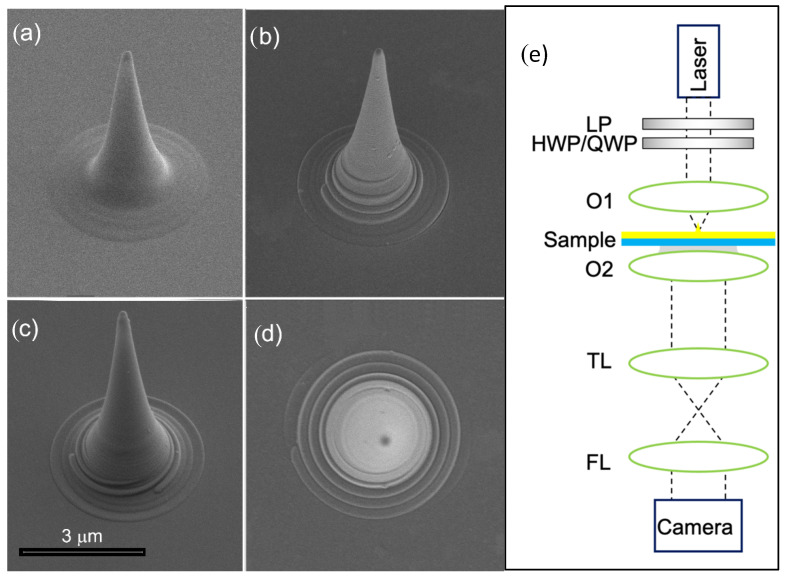
SEM micrographs of the prepared tips. (**a**) Bare tip; (**b**) tip with embedded *m* = 1 spiral; (**c**) tip with embedded *m* = 3 spiral; (**d**) top view of *m* = 1 tip; (**e**) set up of Leakage Radiation Microscopy. The Laser beam’s polarization is controlled by a set of a linear polarizer (LP) and a half or a quarter wave plate (HWP/QWP) and then focused by an objective O1 (details in the text). The imaging objective O2 extracts the leakage radiation through an index-matching oil, which then passes through a tube lens (TL) and a Fourier lens (FL) to obtain the k-space image.

In our experiment, we utilize the fabricated helical tips as generator of directional surface plasmon polaritons in the near field. To measure this, we illuminated the tip by a CW diode laser at $\lambda_{0}=780\;\mathrm{nm}$. The beam was collimated and focused onto our sample by a 20× objective (NA = 0.45) (Olympus, Tokyo, Japan). The near-field SP distribution was imaged by collecting the leakage radiation using an oil-immersion 100× objective (NA 1.25) (Olympus, Tokyo, Japan) that was brought into a contact with the sample substrate. This leakage radiation microscopy system (LRM), described in several papers [12,18,31,32], provides us with a direct image of the plasmonic modes excited at the metal−air interface. By setting an additional lens at the end of the optical path, we were able to access the k-space image of the plasmonic propagation (see Figure 1). The k-space imaging allows us to visualize the iso-frequency surface corresponding to the exciting laser wavelength and also analyze the polarization-dependent effects. To do this, we utilize a linear polarizer (LP) followed by a quarter-wave plate (QWP) or a half-wave plate (HWP) in order to alter the incident state.

## 3. Results

Scanning electron microscope (SEM) images of the prepared tips are reported in Figure 1. As can be seen, the bare metallic tip (Figure 1a) can be easily modified with a chiral element of different topology.

The aforementioned k-space imaging LRM optical setup is depicted in Figure 1e. The tip’s axis was accurately aligned with the optical axis by using nanometric piezo actuators (Thorlabs, Newton, NJ, USA).

**Figure 2:**
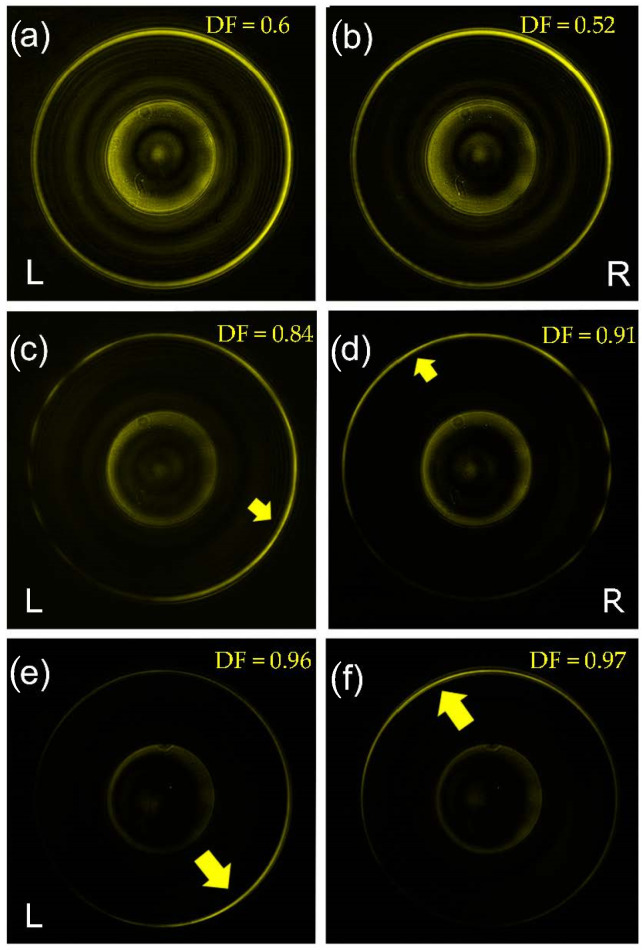
Measured intensity distribution in the k-space from the nanotips for R and L polarizations incident light; (**a**) and (**b**) symmetric tip; (**c**) and (**d**) *m* = 1 tip; (**e**) and (**f**) *m* = 3 tip. Directionality Factor appears in each panel.

Here, we investigate the resonance behavior of the plasmonic signal with respect to the incident polarization. Figure 2 shows the intensity distributions in the k-space for incident right-handed and left-handed circular polarizations (R and L, respectively). In all the cases, the central disk represents the NA of the illumination objective O1. At a distance $k_{\rho }=k_{sp}$, we observe the plasmonic resonance line corresponding to the SP dispersion on a flat surface, namely, $k_{sp}=\sqrt{\epsilon_{m}/\left(1+\epsilon_{m}\right)}$, where $\;k_{0}=2\pi /\lambda_{0}$ and $\epsilon_{m}$ is the dielectric constant of gold at $\lambda_{0}$. We note an important difference between the completely symmetric tip, Figure 1a,b, and the chiral tips with different topologies (*m* = 1 in Figure 2c,d, and *m* = 3 in Figure 2e,f. In the first case, the cylindrical symmetry of our scatterer (the tip) does not introduce any visible asymmetry in the intensity distribution along the azimuthal direction. Moreover, the same intensity profile can be observed for R and L polarizations. This observation is in agreement with theoretical models [26,27]. An asymmetric distribution, and a consequent symmetry breaking and directional plasmon excitation, can be obtained with a misalignment of the tip with respect to the optical axis as previously demonstrated [12]. As we can see from Figure 2, the same effect can be obtained by introducing an asymmetry in the scatterer itself, i.e., by adding a helical corrugation to the tip. As clearly shown in Figure 2c–f, the circular SP resonance line exhibits a strong asymmetry in the intensity distribution along the azimuthal direction. Moreover, we notice that this variation depends on the incident light polarization handedness. Finally, it seems also clear that a higher topological charge (*m*) of the spiral enables a more significant directionality in the excitation. We define a figure of merit for the SP directionality by a directionality factor, $DF=\frac{I_{max}-I_{min}}{I_{max}+I_{min}}$, where $I_{max/min}$ is the maximum (minimum) value of the intensity distribution along the SP resonance circle. In Figure 2, we observe a strong increase in the directionality with the topological order of the spiral groove.

As has been previously discussed [12], the observed helicity-dependent k–space distributions can be modeled as the interaction between broadband phenomena, namely, light scattering from the tip, and the light that was coupled to the SP. It was proposed that the SP plasmon resonance could be represented by a Fano lineshape $I_{F}\;\left(k_{\rho },\;\varphi \right)$ given as [12]:

(2) $$I_{F}(k_{\rho },\varphi )\propto {\left|\frac{q\left(\varphi \right)k_{SP}^{\prime \prime }}{(k_{\rho }-k_{SP}^{\prime })+ik_{SP}^{\prime \prime }}+1\right|}^{2}$$

where $k_{SP}={k^{\prime }}_{SP}+i{k^{\prime \prime }}_{SP}$ is the plasmonic field complex wave number. According to our earlier studies the excitation of the SP is mainly contributed from the light scattering at the subwavelength cone tip [18]. In Equation (2), this broadband scattering is represented by unity while the coupling of the scattered light to the plasmonic wave is represented by the complex number *q*, defined hereafter as a coupling factor. 

This coupling factor depends on the momentum matching between the interacting components and accordingly can vary azimuthally. It has been shown that the phase mismatch can occur due to the excitation of various multipole modes at the scatterer. Additionally, in this case, the strength of the interaction is represented by azimuthally varying coupling factor, q(φ). In the axially symmetric system, the fields are most conveniently described by using the Jones vector for longitudinal and radial components as $E=E\hat{a}$, where $\hat{a}=\left[a_{\rho },a_{z}\right]$ and $a_{\rho },a_{z}$ are some complex numbers. Our tip can then be regarded as a dipole-like emission due to the focused Gaussian beam. When the incident light is circularly polarized, the scattering can be described by the combination of the longitudinal and a rotating radial dipole, namely: ${\hat{a}}_{scat}=[p_{\rho }e^{\pm i\varphi },p_{z}],$ where $p_{\rho }$ and $\;p_{z}$ are normalized dipole component amplitudes. The excitation of a spin-dependent helical phase front in various optical systems is well described in terms of the spin–orbit interaction and has also been studied already for plasmonic systems [14,23,24,25,26,27,28,33]. 

The plasmonic field vectorial structure is given as ${\hat{a}}_{SP}=\frac{1}{\sqrt{1+\chi^{2}}}\left[\begin{array}{cc} i\chi , & 1 \end{array}\right]$ so the coupling factor depends on the overlap between the exciting scattering field and the SP wave as $q\left(\varphi \right)\propto \;{\hat{a}}_{SP}|{\hat{a}}_{scat}$. Accordingly, for a perfect coupling, the quarter period phase lag is required. Nevertheless, when the normal component of the scattering field, *p_z_*, is nonzero, this phase matching can be only achieved in a specific azimuthal direction *ϕ *. This is where one can observe the maximum in the plasmonic resonance ring in the k-space. For purely symmetric structures and an accurate alignment of the sample with the beam axis, the normal component vanishes and the SP distribution is uniform as in Figure 2a,b. We believe that by introducing the helical groove on the tip’s surface, we excite a z-dipole that breaks the symmetry in a spin-dependent fashion and leads to the directionality observed in Figure 2c–f. 

Following these observations of spin-dependent SP behavior, we decided to investigate the circular dichroism induced by the chiral tips. As is well known, chiral structures exhibit optical activity manifested by the differential absorption of circular light states [34]. This effect leads to a measurable ellipticity of the incident linearly polarized light and can be directly derived by separately measuring the transmission of circular polarization states as follows:

(3) $$\Delta =tg\left(\chi \right)=\;\left(\sqrt{I_{R}}-\sqrt{I_{L}}\right)/\left(\sqrt{I_{R}}+\sqrt{I_{L}}\right)$$

where *I_R_* and *I_L_* are the intensities for right and left polarization, respectively, and χ is the ellipticity angle. This way a spatial CD spectrum can be obtained. In contrast with a most common temporal CD (frequency-dependent ellipticity variation), the results presented here show k-space maps of the ellipticity. Clearly, the most significant contributions to these maps appear at the central part of the k-space due to the light scattering and from the SP resonance circle at $k_{\rho }=k_{SP}$.

**Figure 3:**
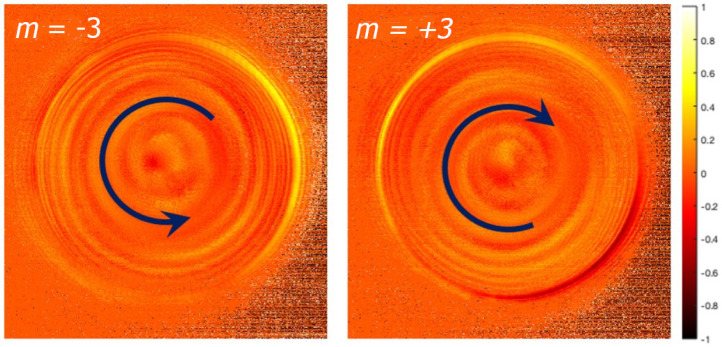
Measured circular dichroism of the tips with m=±3.

Considering the better directionality obtained with the tips with *m* = 3, we used them to measure the CD (for left-handed and right-handed spiral). Figure 3 reports the measured CD maps. One can notice a very clear helical structure of the measured maps with opposite handedness, which we link to the strong symmetry breaking by the tips. In chiral system, two enantiomers can be interconverted by a spatial inversion rather than by a time reversal [34,35]. As we observed, also the k-space CD maps geometrically behave as two enantiomers. Therefore, here the CD spectra of the positive and the negative *m* were compared.

Although this signature is still under study, the most important conclusion here is that our subwavelength spiral groove fabricated on a nanoscale taper produces a macroscopic measurable pattern enabling one to distinguish between different light illumination states.

## 4. Conclusions

In summary, we experimentally observed a strong directionality of plasmonic waves excited by a chiral nanotaper resulted from a spatial Fano-like effect. The effect becomes evident when investigating the leakage signal in the k-space, where the interplay between the resonance and the anti-resonance is clearly visible. The variation of the plasmonic coupling efficiency occurs due to the symmetry breaking of the structure leading to the non-zero contribution of the z-dipole component of the tightly focused field. We have proposed a robust fabrication method of chiral nanoscale Au tips for plasmonic polarization-dependent excitation. Several structures with different topologies and handedness have been studied under the illumination of a circularly polarized beam. We have also observed a spiral shape of the measured circular dichroism k-space map whose handedness was consistent with the tip structure. This led us to a conclusion that the observed Fano effect is highly sensitive even to subwavelength structure symmetry breaking, which opens the avenue for future nanophotonics applications in sensing and biophotonics. 

## Data Availability

Data is contained within the article.
